# Religion and Fertility in Western Europe: Trends Across Cohorts in Britain, France and the Netherlands

**DOI:** 10.1007/s10680-015-9371-z

**Published:** 2016-01-28

**Authors:** Nitzan Peri-Rotem

**Affiliations:** Department of Sociology, University of Cambridge, Free School Lane, Cambridge, CB2 3RQ UK

**Keywords:** Religion, Fertility, Transition to first birth, Western Europe, Secularization

## Abstract

The role of religion in explaining fertility differences is often overlooked in demographic studies, particularly in Western Europe, where there has been a substantial decline in institutional forms of religious adherence. The current study explores the changing relationships between religion and childbearing in Britain, France and the Netherlands. Using data from the Generations and Gender Programme and the British Household Panel Survey, religious differences in completed fertility and the transition to first birth are explored across cohorts of women. In addition, a longitudinal analysis is employed to examine the influence of religion on subsequent childbearing. Although the secularization paradigm assumes that the influence of religion on individual behavior will diminish over time, it is found that religious affiliation and practice continue to be important determinants of fertility and family formation patterns. However, there is some variation in the relationship between religion and fertility across countries; while in France and the Netherlands fertility gaps by religiosity are either consistent or increasing, in Britain, this gap appears to have narrowed over time. These findings suggest that fertility differences by religion also depend on the particular social context of religious institutions in each country.

## Introduction

Throughout the past decades, most developed countries have experienced substantial transformations in fertility and family formation patterns. These changes, which include delays in first marriage and first birth, as well as a sharp decrease in fertility rates, are often associated with the process of secularization and the shift of values toward greater individualization (Goldscheider 2006; Lesthaeghe and Surkyn 1988; Norris and Inglehart 2004; Surkyn and Lesthaeghe 2004).

The standard theory of secularization contends that processes of modernization, including advancements in science, technology, education and economic development, would result in gradual erosion in the importance of religion, until it loses its significance in social and private life (Bruce 2011; Wilson 1966). This process is assumed to have a major influence on changing family behaviors, since decisions about union formation and reproductive choices are increasingly guided by personal aspirations of self-fulfillment, rather than by the moral order of religious institutions (Lesthaeghe 2010; Lesthaeghe and Surkyn 1988; van de Kaa 1993).

The declining significance of religion in society has also been a common explanation to the apparent convergence into lower fertility levels among different religious denominations in the USA and in Ireland (Goldscheider and Mosher 1991; Mosher et al. 1992; O’Grada and Walsh 1995; Westoff and Jones 1979). In particular, there has been a marked decline in fertility levels among those identified as Catholics. As a result, Catholic fertility became closer to the fertility levels of other major religious denominations, a process that was described as “the end of Catholic fertility” (Westoff and Jones 1979). The narrowing fertility gap between Catholics and other denominations, alongside the secularization paradigm, has led to a diminishing interest in the role of religion in explaining demographic behavior (McQuillan 2004; Philipov and Berghammer 2007). In particular, the relationship between religion and fertility received little attention in research on Western European countries, which are considered to be highly secularized (Inglehart and Norris 2003).

It should be noted, however, that the classic theory of secularization has been widely criticized by numerous scholars, who have claimed that the decline in religion is neither as widespread nor consistent as previously assumed (Davie 1990, 2007; Greeley 2003; Norris and Inglehart 2004). According to Greeley (2003), no common pattern of secularization could be found in Europe; while in some countries religion has declined, in others it has remained relatively unchanged or even increased. Although there is a general agreement that the influence of religious institutions on society in Europe has weakened, there is an ongoing debate on the continuing influence of religious ideas on attitudes and behavior of individuals (Voas and Doebler 2011). Furthermore, some scholars have maintained that a general decline in religion does not necessarily imply a weakening effect of religiosity on demographic behavior; rather, the increasing cleavage between secular and religious individuals may have sharpened the differences between these groups in terms of social and moral attitudes, which also concern family behaviors (Kaufmann 2010; Régnier-Loilier and Prioux 2008; Stegmueller et al. 2011).

In the past decade, there has been a renewed interest in the effect of religion on fertility, and recent studies have pointed to the persisting influence of religion on fertility patterns; for example, Frejka and Westoff (2008) found that in Europe and the USA, women who identify as Protestants or Catholics had higher fertility rates compared with women who declared having no religion. Additionally, within each denomination, the more devout—in terms of service attendance and importance of religion in daily life—had larger families. Similarly, in a comparative study of 18 European countries, Philipov and Berghammer (2007) reported a positive correlation between different measures of religiosity (e.g., affiliation, practice and self-rated religiosity), and individuals’ intended and actual fertility. The positive effect of religiosity on family size received further support in a longitudinal study from the Netherlands, showing that church attendance is a strong predictor of future childbearing (Berghammer 2012). In addition, using cross-sectional data from 1985 and 1999 in Spain, Adsera (2006a) has found that fertility differences between practicing and non-practicing Catholic women have grown, since fertility decline occurred only among the latter group.

However, most studies in this topic are based on cross-sectional analysis, which estimate the relationship between religion and fertility in a given point in time. The current study uses both retrospective and longitudinal data to explore whether in the context of a general religious decline, individual religiosity has become a less important determinant of family size or whether there is actually an increasing divergence between religiously active individuals and those with lower attachment to religious tradition. For this purpose, retrospective birth histories of women from Britain, France and the Netherlands are employed in order to follow trends in religious differences in completed fertility and the transition to first birth across cohorts. In addition, a longitudinal analysis is performed to estimate the effect of religious adherence on subsequent childbearing.

This study focuses on two main dimensions of religious adherence: affiliation with a particular denomination and religious practice, as well as the combination between them. Religious affiliation is regarded as a meaningful form of identification, which represents the cultural background into which a person was born or grew up in and may have consequences on social behavior (Day 2011; Southworth 2005). In addition, religious practice—as measured by frequency of attendance at religious services—is another key measure of religious commitment and is considered to be a more reliable indicator of religiosity compared with religious belief or private prayer, since it has a public aspect to it and it involves the investment of time and effort (Storm and Voas 2012; Voas 2009). Furthermore, it is important to pay attention to the interdependencies between religious affiliation and practice; first, the intensity of religious involvement is expected to accentuate the effects associated with religious affiliation (Lehrer 2004a), and second, different religious traditions have contrasting expectations about the frequency of religious service attendance (Voas and Doebler 2011). For example, Sunday mass attendance is considered an obligation by canon law for Catholics, while for Protestants, instructions regarding church attendance are more relaxed, although it is still strongly encouraged (Bruce 2011). Nonetheless, church attendance rates may differ across countries and over time (Greeley 2003; Hout and Greeley 1987; Inglis 2007).

As a result of immigration trends, a growing proportion of people in Western Europe are affiliated with a religion other than Christianity, with Muslims being the largest group among them (Pew Research Center 2009). As a whole, those identified as Muslims tend to be more religious and also to have higher fertility rates compared with the Christian majority (Kaufmann et al. 2012; Régnier-Loilier and Prioux 2008). It should be noted, however, that non-Christian groups form less than 10 % of the population in the countries observed in this study (Pew Research Center 2012), and sample sizes for these groups are relatively small. Therefore, the main focus of the current research is on fertility trends among Christian denominations and the continuously growing population of non-affiliated individuals.

The remainder of the article is structured as follows: Sect. 2 provides an overview of recent empirical findings and theoretical explanations for the influence of religion on reproductive behavior. Section 3 includes a description of religious indicators and sociocultural context of the countries selected for this study. Against this background, the research hypotheses about the relationship between religion and fertility and the way it changes across cohorts are formulated. Then, Sect. 4 provides details on the data sets, measures and empirical procedure. This is followed by the presentation of findings in Sect. 5. Section 6 concludes with a summary and discussion of findings and the implications for future research.

## Religion, Religiosity and Fertility Trends

Theoretical explanations for the effect of religion on fertility behavior stress the high value that most religions ascribe to family and children, alongside other fertility-related doctrines, which emphasize women’s familial roles (Lehrer 2004a; McQuillan 2004; Norris and Inglehart 2004; Sherkat 2000). It should be noted, though, that some denominations may put greater emphasis on childbearing than others. For example, the higher fertility of Catholics in comparison with Protestants was often seen as a consequence of pro-natalist Catholic teachings that forbade the use of artificial means of contraception (McQuillan 2004). Furthermore, the Catholic Church has a strict opposition to divorce, since marriage is seen as a lifelong, unbreakable commitment between men and women and as the sacred basis for family formation (Richards 2009). While Protestant Churches also promote traditional family values and the procreation of children as the main purpose of marriage, the approach of Protestant traditions toward the use of contraception tends to be more pragmatic (Creighton 2009; McQuillan 2004). However, it has been argued that adherence to church teachings on contraceptive use among Catholics has substantially weakened over time and that this may account for the sharp decrease in Catholic fertility rates and the convergence of family size between Catholics and other denominations (Goldscheider and Mosher 1991).

According to McQuillan (2004), specific social and political settings may determine the extent to which religion influences fertility patterns; for example, religious groups are expected to have a greater effect on behavior when members feel a strong sense of attachment to the religious community, or when it is considered to be an important marker of identity. Furthermore, religious norms about family and fertility are most likely to influence behavior when religious institutions have the means to communicate these teachings to their members and to enforce compliance, through formal organizations or informal social pressure. Thus, religious service attendance is assumed to both reflect and reinforce belief and commitment to traditional religious values, through the repeated exposure to religious teachings and interaction with people who share similar values (Davie 2007; McQuillan 2004).

Another route through which religious participation may influence fertility is through increased social capital among those attending religious services on a regular basis; previous studies have found that religious congregations promote the formation of social networks, where different types of informational, emotional and practical support are exchanged between members (Chatters and Taylor 2005; Philipov et al. 2006; Putnam 2000; Waite and Lehrer 2003). This source of support may affect positively on fertility decisions by reducing uncertainty and by lowering the perceived costs of childbearing. Furthermore, religious belief and practice can contribute to coping with stressors and difficulties of daily life, including those related to family formation and expansion (Chatters and Taylor 2005; Krause et al. 2001; Pargament et al. 2000). For these reasons, religious involvement and church attendance in particular is expected to promote higher fertility.

These explanations are in line with empirical studies which show that religious service attendance is a stronger predictor of fertility intentions and behavior, compared with affiliation alone (Adsera 2006a, b; Philipov and Berghammer 2007). Moreover, Adsera (2006b) has argued that following the declining influence of religious institutions in Europe, church attendance has become a more salient determinant of fertility norms among younger generations, as those who continue to go to church represent a more selective group of people who adhere to religious doctrines. A similar process is described by Davie (2007), who contends that church attendance has increasingly become a matter of personal choice, leaving a small minority of religiously active people, although with strong attachment to religious convictions. Therefore, over time, the underlying differences in fertility behavior between practicing and non-practicing individuals may become more pronounced (Kaufmann 2010; Régnier-Loilier and Prioux 2008).

## Religious Indicators in Britain, France and the Netherlands

The three countries at the focus of this study are considered among those that have undergone the most notable religious decline in Europe over the past century (Greeley 2003; Norris and Inglehart 2004). This is evident mainly by the generational decline in the proportion of religiously affiliated individuals and in the number of people who attend religious services regularly, especially among the cohorts born after the Second World War (Greeley 2003; Inglehart and Norris 2003; Voas 2009).

Nevertheless, these countries greatly differ from one another in terms of their religious heritage and distribution of religious groups. The religious landscape in Britain and the Netherlands is relatively diverse and comprised of different Protestant denominations alongside a substantial Catholic population. In France, on the other hand, Roman Catholicism forms the dominant religion; in 2010 the share of baptized Catholics of the total French population was estimated at 75 % (Pew Research Center 2013). By contrast, Britain has a Protestant majority, where the Church of England is the largest religious denomination, forming about a quarter of the population (Voas and Ling 2010). Roman Catholics are the second largest group, forming around 10 %, while most other Christians belong to various reformed groups (ibid). Thus, over half of the population in Britain are self-identified Christians (NRS 2013; ONS 2012). In the Netherlands, the religious divide between Protestants and Catholics is even further pronounced. As part of the pillarization system, which prevailed from the end of the nineteenth century to the mid-twentieth century, members of each denomination, or unaffiliated people, belonged to separate social, educational and political institutions (van Poppel 1985). However, the pillarization system has dissolved since the 1960s, as secularization accelerated and the proportion of disaffiliated people has increased rapidly (Knippenberg 1998; van Rooden 2003). Today, Catholics constitute around 30 percent of the population in the Netherlands, while Protestants constitute about a fifth (Berghammer 2012). The Protestant population is rather heterogeneous and is comprised of mainline Dutch Reformed, as well as more conservative groups, including Orthodox Calvinists and Evangelical denominations (Knippenberg 1998).

While the majority of people in the countries observed here identify themselves as affiliated with a particular religion, only a minority attend religious services on a regular basis. Close to a fifth of the population in Britain and the Netherlands attend religious services at least once a month while just over a tenth of the French population do so (Davie 2002; Statistics Netherlands 2012; Voas and Ling 2010).^1^ However, attendance rates vary greatly across religious denominations. For example, church attendance rates are markedly higher among British Catholics than among those affiliated with the Church of England (40 % of Roman Catholics attend at least once a month compared with 18 % of Anglicans, see: Voas and Ling 2010). In the Netherlands, attendance rates are currently higher for Protestants than for Catholics, due to the dramatic fall in participation rates among the latter; while more than 70 % of Roman Catholics were regular church goers in 1970, this figure has dropped to less than a quarter by the end of the twentieth century (Knippenberg 2005). Across the same period, regular attendance among Dutch Reformed has dropped from 50 to 40 %, and among other Protestant streams (e.g., Orthodox Calvinist) about two-thirds remained regular attenders, following a drop from nearly 90 % (ibid).^2^ These trends may be the result of the traditional divide between liberal and fundamentalist wings within the Dutch Reformed Church; on the one hand, resignation from the church has started as early as the 1880s, while on the other hand, the fundamentalist wing may have contributed to maintaining relatively high participation rates (Knippenberg 1998, 2005).

In France, religious participation rates have been and continue to be considerably lower compared with those in other Catholic countries, such as Spain, Italy or Ireland (Davie 2002; Norris and Inglehart 2004). This may be partly attributed to the historical conflict between church and the state in this country and the relatively strong anti-church sentiments among the French people (Greeley 2003; Martin 2005). Nonetheless, recent data indicate that the low rates of church attendance among French Catholics have reached a plateau and remained stable over the past decade (Pew Research Center 2013).

Compared with those identified as Christians, religious participation among members of other religions (most of whom are Muslims) is expected to be higher, since this group includes a larger proportion of immigrants from highly religious countries (Kaufmann et al. 2012). Moreover, religion may play a more significant role in the social identity of minority groups (Fetzer and Soper 2005; Southworth 2005).

As only a minority of the population in Western Europe attend religious services, these countries consist of a large proportion of ‘nominal’ religious people, who identify as affiliated with a specific denomination, but do not attend religious services on any regular basis. This phenomenon has been described by Voas (2009) as “fuzzy fidelity,” since nominally affiliated individuals are neither religious nor completely secular. Moreover, Voas maintains that this is a transient stage toward increasing disaffiliation from religion. Some scholars have argued that religious identification is becoming less a matter of adherence to church teachings and regulations, and more a matter of belonging to a shared cultural heritage (Hervieu-Léger 1990, 2000; Inglis 2007; Pace 2007). This may especially be the case within Catholicism, as increasing numbers of self-identified Catholics do not share the views of the Catholic Church leaders on sexuality, which may also account for the fall in attendance (Hout and Greeley 1987; Pace 2007). Similarly, Day (2011: 72) has concluded that many people identify as Christians simply because they were baptized or attended church when they were younger, or since it is closely associated with national or ethnic identity (Day 2011; Voas and Bruce 2004). However, compared with practicing religious people, nominally religious individuals are less likely to consider religion as an important source of guidance in everyday lives (Day 2011; Voas 2009).

Under these circumstances, as the proportion of regular church attendance is shrinking from one generation to another, we may expect this group to show increasingly distinct patterns of fertility and family formation, as they are more likely to continue adhering to traditional religious doctrines that highlight the value of family and children, alongside the fulfillment of traditional family roles (Adsera 2006a, b; Goldscheider and Mosher 1991). Moreover, behavioral features of the second demographic transition, including the delay in first birth and the shift to below replacement fertility, are less likely to appear among those individuals who are dedicated to traditional forms of religious practice (Surkyn and Lesthaeghe 2004).

Therefore, it is first hypothesized that *individuals who attend services on a regular basis (at least once a month) will have larger family size compared with those who attend services less often or not at all*. The second hypothesis postulates that *nominally affiliated individuals (those identifying with a particular religion but do not attend services on a regular basis) would have smaller family size compared with religiously active individuals, though larger than those who stated having no religious affiliation*.

The third hypothesis contends that *fertility differences between regular and non*-*regular service attendants will increase among younger birth cohorts*. Since the first group is becoming smaller and more selective, those women with stronger adherence to religious doctrines are expected to maintain high levels of fertility, while other women would experience fertility decline.

In this context, women with lower attachment to religious tradition would have increasingly higher likelihood of remaining childless. Therefore, the fourth hypothesis states that *more religious women, in terms of affiliation and practice*, would *be more likely to experience the transition to first birth and that this gap will increase among the younger birth cohorts*. The following section provides a description of the data sources and methodology that are used to examine these propositions.

## Data, Measurement and Methods

### Data and Samples

The data sources used for this study are the British Household Panel Survey (BHPS) (University of Essex, Institute for Social and Economic Research 2010) and the Generations and Gender Programme (GGP) surveys for France and the Netherlands (United Nations 2005). The BHPS is designed as an annual survey of a nationally representative sample of over 5000 households. The same individuals aged 16 and above were interviewed in successive waves since 1991 through 2008. The BHPS survey is complemented with additional data from the consolidated union and births histories (Pronzato 2011), which contains retrospective lifetime histories and subsequent panel data related to respondents’ partnerships and childbearing. The descriptive data for Britain are based on the last wave of the panel survey, which was conducted in 2008 on a sample of nearly 12,200 respondents.

The GGP is a system of nationally representative longitudinal surveys, coordinated by the United Nations Economic Commission for Europe (UNECE). These surveys include detailed information on partnership and birth histories, as well as socioeconomic variables and data on religious affiliation and service attendance. The French survey was first conducted in 2005 on a sample of over 10,000 respondents, of which around 6500 were interviewed again in 2008. The survey from the Netherlands was conducted between 2002 and 2004, using a sample of 8161 respondents, and around 6100 of the original sample were re-interviewed in 2006–2007. Due to the large proportion of attrition between the two waves, the descriptive analyses for France and the Netherlands are based on data from the first wave. For the longitudinal analysis, which incorporates both waves of the panel, a propensity score is used to control for possible bias as a result of non-random attrition (see “Appendix”).

### Measures

#### Religion

In both the BHPS and the GGP, respondents are asked to name a particular religion to which they adhere. In all three countries, respondents could choose from a list of country-specific religions, including specified Christian denominations and a category of “no religion.” It should be noted, though, that there were some differences in the phrasing of this question in each country. The question in the BHPS is phrased as: “Do you regard yourself as belonging to any particular religion? If Yes: Which?” The parallel question in the Netherlands is phrased as: “Do you consider yourself to belong to a particular faith, religious denomination or church? If so, which one?”, while the question in France is phrased in a more affirmative manner: “What is your religious affiliation (or your religion by birth)?”^3^ These differences in phrasing may have some effect on respondents’ answers to this question; it has been argued that answers to questions on religion may be susceptible to factors such as the context in which the question appears on the survey, the way it is formalized and even more importantly, by the social and political context in a given place and time (Voas and Bruce 2004). Therefore, cross-national comparisons based on religious affiliation in each country should be interpreted with due caution.

Religious practice was measured by the frequency of attendance at religious services. In Britain and France, the question is practically identical (“How often, if at all, do you attend religious services?”), with five possible answer categories in Britain: “once a week or more,” “at least once a month,” “at least once a year,” “never” and “only weddings, funerals, etc.” In France, respondents could answer with a specific number in time units of a week, a month or a year. In the Netherlands, the question is phrased in a similar manner and has the same answer categories as in Britain apart from the fifth option for special events.

Since the study aims to differentiate between regular and non-regular attendants at religious services, religious practice was dichotomized to those attending once a month or more (“practicing”) and those who attend less often or never (“nominal”). According to Burkimsher (2014), monthly attendance is a commonly used cutoff between attenders and non-attenders, although some studies use weekly attendance. Since those attending services on a weekly basis form a small sample size in some denominations, monthly attendance was the preferred cutoff point. Furthermore, since monthly attendance may have different meaning in different denominations, a combined religious variable was constructed, so respondents were divided into “practicing” and “nominal” within each denomination. Those who stated they have no religion were classified under the “no religion” group.

#### Education

The measure for education is based on the International Standard Classification of Education (ISCED 1997) to enable cross-country comparison. The six education categories of the ISCED were grouped into three levels: “lower secondary,” which refers to less than completed secondary school, “upper secondary,” which refers to completed secondary school, or any post-secondary education that is non-tertiary. The highest level, “tertiary education,” refers to Bachelor or higher degree.

#### Nativity

Since immigrants tend to have both higher rates of religious participation and differential fertility behavior (Burkimsher 2014; Kaufmann et al. 2012), a binary variable that indicates whether or not the respondent was born in the country of interview is included in the analysis.

### Analytical Strategy

As the sample design and religious structure differ among the observed countries, all analyses are conducted for each country separately. In addition, the descriptive analyses were calculated using country-specific population weights. The first part includes the religious distribution in each country (including non-affiliation) across 10-year birth cohorts from 1930 through 1979. The proportion of regular attendants in each denomination and their share of the total population by cohort are also presented. The minority religions (e.g., Muslims, Buddhists, Sikhs and Jews) were collapsed into ‘Other’ religions. However, since the sample size for this group is too small to enable meaningful exploration of fertility trends across cohorts, they are not included in all other analyses.

The next part examines trends in completed (or near completed) family size^4^ by religious group (the combined religion and practice variable) across subsequent cohorts of women starting from the 1930 through 1965, which is the most recent cohort with near completed fertility data. A multivariate regression analysis is then employed to test for fertility differences between nominally and practicing religious women to non-affiliated ones. In addition, interaction terms between birth cohort and religious group are included, to examine changes in this relationship between earlier and recent cohorts. Then, an event history model is employed to analyze differences among religious groups in the transition to first birth. In this model, the births and partnership histories of each woman are reconstructed from the age of 15 to the age of 45, and the cases in which a woman did not give birth by the age of 45 or until the last date of interview were right censored. Based on this model, the median age at first birth and the proportion of women who remained childless at the age of 45 were obtained. In addition, the share of first births outside marriage was calculated for each religious group in order to learn about the interdependencies of marriage and first birth among these groups. These analyses are done separately on a subsample of women who were born before 1960 and those born afterward, in order to observe religious differences in first birth patterns among women from earlier and more recent cohorts.

A discrete-time hazard model for the transition to first birth is then performed separately for women born before or after 1960.^5^ This model is estimated using a multivariate logistic regression analysis, where the probability of experiencing the transition to first birth is calculated for each woman-year as a function of both fixed and time-varying covariates. Thus, the logistic model can be formalized as follows:

$$\log \left[ {\frac{P\left( t \right)}{1 - P\left( t \right)}} \right] = \alpha + \sum_{j} \beta_{j} x_{j} + \sum_{i} \beta_{i} x_{i} (t)$$

where log[*P*(*t*)/1 − *P*(*t*)] is the log of the odds for the occurrence of first birth in a given year (*t*). The *α* represents the constant term, *x*_*j*_ are fixed-time variables and *x*_*i*_(*t*) denotes a set of time-varying covariates whose values may change from one year to another. The set of fixed-time variables includes the combined religious group variable, highest level of education achieved and nativity (as defined above). The time-varying covariates include marital status, which is comprised of four categories: single, in cohabitation, married and divorced/separated/widowed. In the GGP data, each respondent was asked about previous relationships and the month and year they started living together with each partner. Where applicable, the time of formal marriage or end of union was also recorded. In the Netherlands, however, the time of previous marriages is not recorded. Instead, the survey includes information on the time the couple started living together and whether they were married or not. Therefore, for this country, the start year of cohabitation in previous partnerships is also used as a proxy for the timing of marriage. In the BHPS, the information on union history is based either on data from the panel or on retrospective questions about previous unions. The start and end dates for each cohabitation and marriage are also available in this data set. In some cases, however, the start date information is missing, and therefore, these observations were excluded from the analysis. Since women may defer family formation until after they complete their education (Blossfeld and Huinink 1991; Ní Bhrolcháin and Beaujouan 2012), the model also incorporates a time-varying covariate of educational enrollment. This is a dummy variable—based on respondents’ age when leaving the educational system—which indicates whether a woman is currently enrolled in education or not. In addition, the model controls for age (including age squared) and 5-year time periods.

Nonetheless, it is difficult to draw conclusions about a causal relationship between religion and fertility when using retrospective data, since indicators of religious adherence are measured only at the time of interview, after childbearing has already occurred (Marcum 1988). Furthermore, several studies from the USA have found that life-cycle events, such as marriage and childbearing, are associated with a subsequent increase in religious participation (Ingersoll-Dayton et al. 2002; Stolzenberg et al. 1995; Thornton et al. 1992). Although little or no evidence of family formation effects on religiosity was found in similar studies from Europe (Berghammer 2012; Tilley 2003), there is still a risk of reverse causality in the religion–fertility relationship.

Since the GGP surveys from France and the Netherlands include questions on religious affiliation and practice only in the first wave of the panel, it was not possible to perform a comparative analysis of life-cycle effects on religiosity. However, a longitudinal analysis could be used to examine the role of religiosity as a determinant of future childbearing in each country. This was done by estimating the likelihood of women aged 20–42 with at least one child to proceed to a higher parity within a period of 3 years. Two panel waves are used in each country, covering the years 2004–2007 in Britain,^6^ 2005–2008 in France and 2002–2007 in the Netherlands. A logistic regression analysis is used to predict whether an additional birth occurred between the first and the second wave as a function of women’s religious affiliation and practice in the first observation. The time in years that elapsed since the last birth and the second time the woman was interviewed is included in the model, to control for differences in birth intervals. In addition, the model controls for other indicators, including age, parity, marital status, education and employment status at the time of the first wave.

## Results

### Trends in Religious Affiliation and Practice

**Fig. 1:**
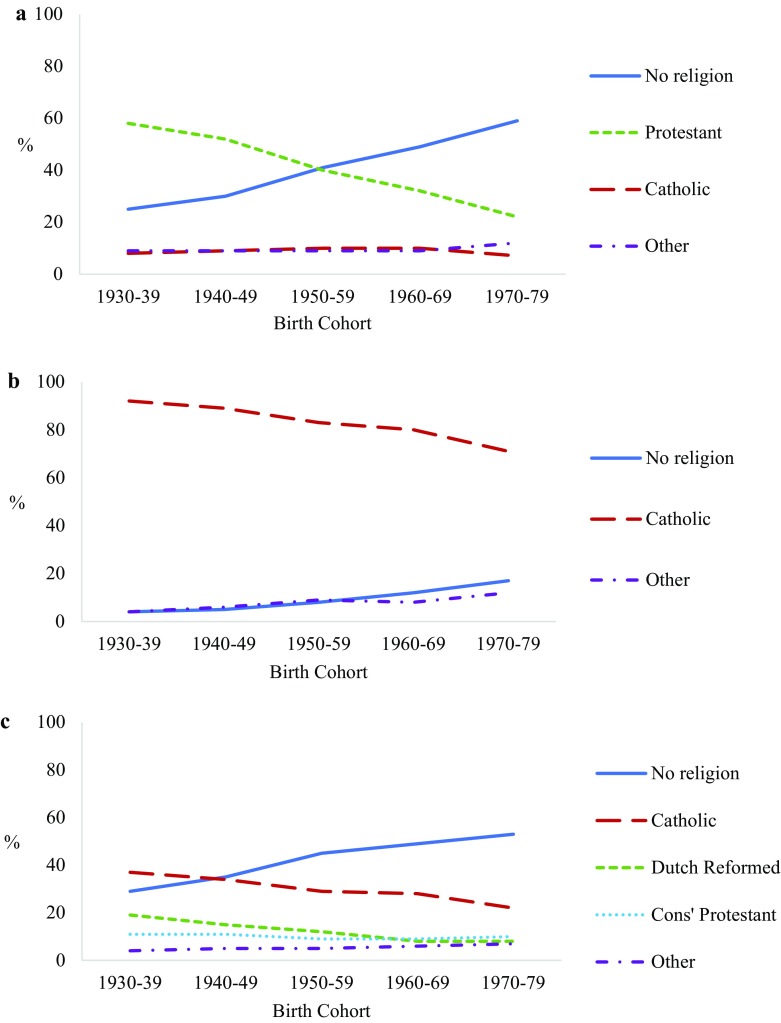
Religious affiliation by birth cohort with country-specific population weights. **a** Britain, **b** France, **c** Netherlands

Figure 1a–c presents the distribution of religious affiliation by birth cohorts in Britain, France and the Netherlands from 1930 to 1979 (for descriptive statistics and sample sizes, see “Appendix,” Table 5a–f). As in previous studies, the proportion of people who identify as belonging to a particular denomination decreases in each subsequent cohort in all three countries. Both in Britain and in the Netherlands, religiously affiliated people are the majority until the cohorts born in the 1970s, in which the group of “no religion” becomes the majority. Nearly 60 % of the youngest cohort in Britain state they have no religion, while in the Netherlands this proportion is slightly lower (53 %). In France, on the other hand, the proportion of non-affiliated people is relatively low and the vast majority of people define themselves as Roman Catholic, although their share is decreasing from one cohort to another. This may indicate a strong attachment to Catholic cultural identity, which is also associated with national French identity (Byrnes 2005; Hervieu-Léger 1990, 2000; Pace 2007), although this proportion may also be exaggerated as a result of the particular phrasing of the question on religious affiliation.

The increase in the “no religion” group in Britain from one cohort to the next appears to be mainly at the expense of those who identify as Protestants, as their proportion among recent cohorts is closer to that of Catholics. The latter group, on the other hand, remains stable at around 8–10 %. In the Netherlands, the share of those affiliated with the Dutch Reformed Church as well as the proportion of Roman Catholics shows a decrease across cohorts. On the other hand, the proportion of conservative Protestants (Evangelicals and Orthodox Calvinists) remains stable at around 10 %. In all countries, the share of those affiliated with the minority religions is larger among the younger cohorts, although it remains relatively low.

**Fig. 2:**
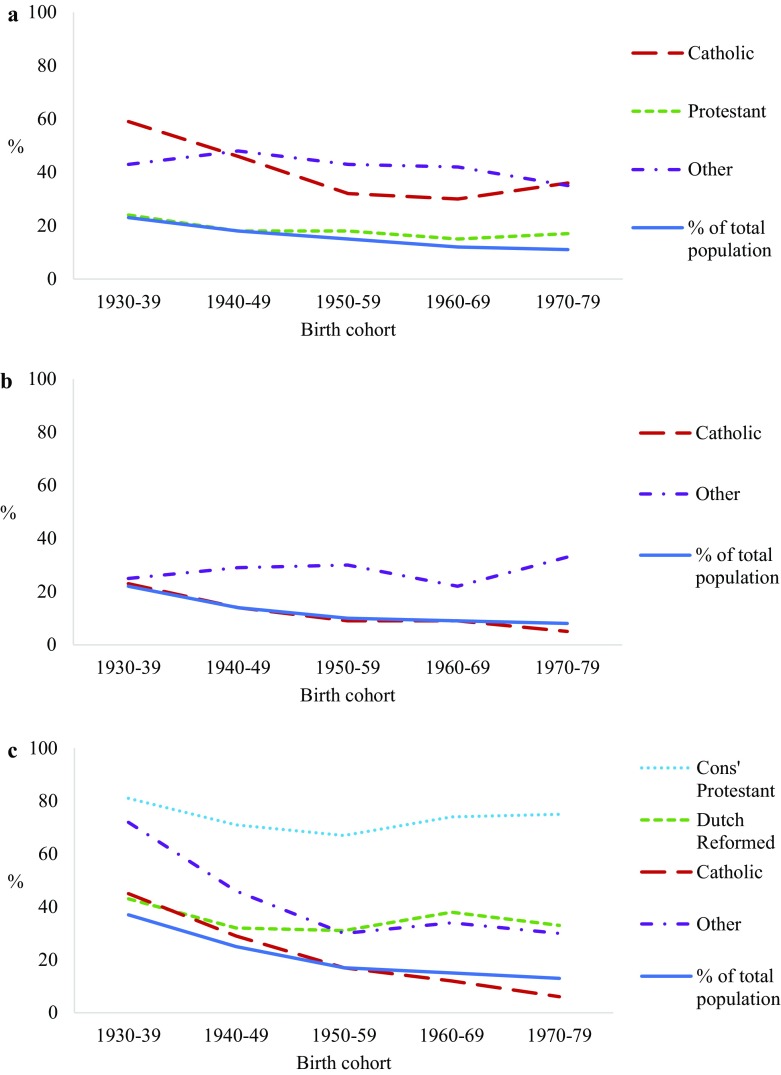
Proportion of regular attendants at religious services by denomination and birth cohort with country-specific population weights. **a** Britain, **b** France, **c** Netherlands

Figure 2a–c shows the proportion of regular attendants at religious services (at least once a month) within each birth cohort and by religious denomination. The solid line indicates the total proportion within the population who are regular attenders.

As in the case of religious affiliation, the share of religiously active people is much smaller among the younger cohorts. While over a fifth of the oldest cohorts in Britain and France are regular attenders, their proportion among the youngest cohort declines to 11 % in Britain and only 8 % in France.

In the Netherlands, attendance rates appear to be slightly higher, with almost 40 % among the oldest cohort, which decreases to 13 % among the youngest cohort. Nonetheless, the rate of decline in each country appears to slow down, as attendance rates for the 1970–1979 cohort are not very different from those who were born a decade earlier. These results are in line with recent studies, showing that the decline in religious service attendance in Western Europe is leveling off among younger cohorts (Burkimsher 2014; Kaufmann et al. 2012).

Furthermore, the decline in religious practice among Catholics appears to be steeper compared with that in other religions. This is similar to the trends found in the USA, which were attributed in part to the growing dissent to the church teachings on sexuality (Hout and Greeley 1987). As in the general population, the group of other religions also show a decline in service attendance among the younger birth cohorts, except in the case of France, where this group shows no consistent trend.

### Fertility Trends by Religiosity

**Fig. 3:**
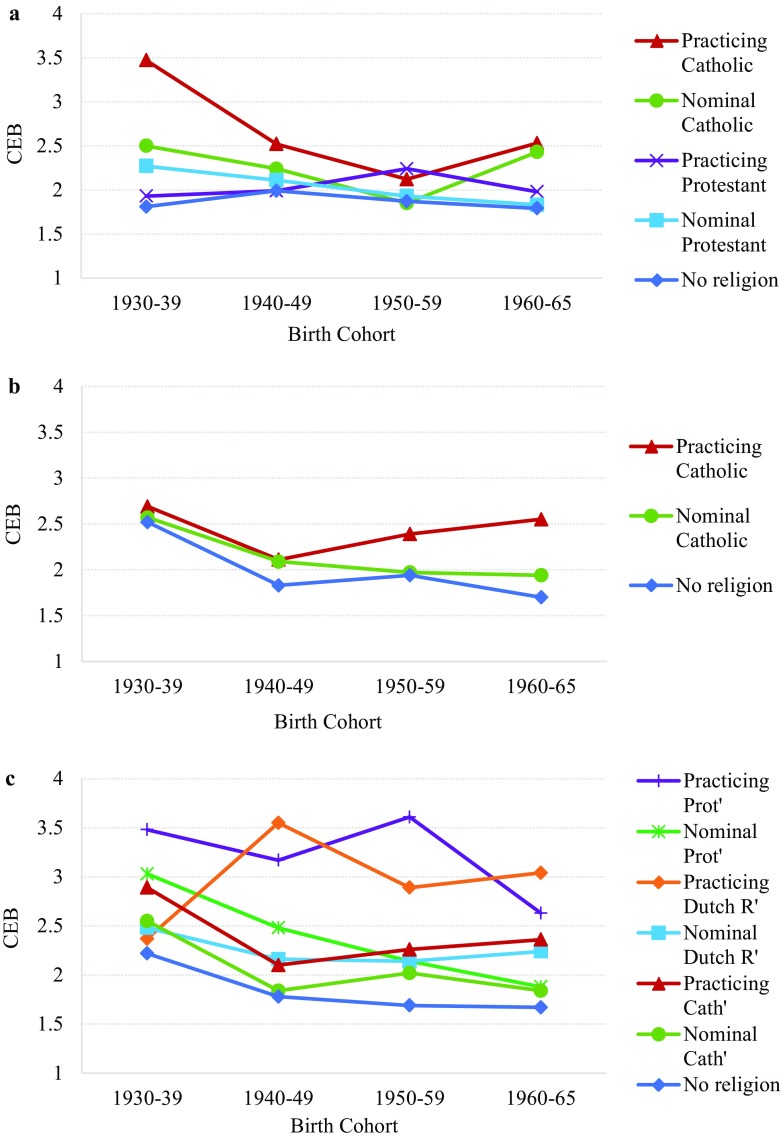
Children ever born by women’s birth cohort and religious group (descriptive data using country-specific weights) [regular attenders within each group are classified as “Practicing” (at least once a month) and non-regular attenders (less than once a month) are classified as “Nominal”]. **a** Britain, **b** France, **c** Netherlands

This section includes findings on trends in completed fertility across birth cohorts of women by religious affiliation and practice. The results from Britain (Fig. 3a) show a sharp decline in fertility among practicing Catholic women from 3.5 children on average among those born in 1930–1939 to around replacement level in the 1950–1959 cohort and then an increase to 2.5 among the youngest cohort. Nominal Catholic women experienced a similar trend with a decline from 2.5 children to 1.9 and then a rise to 2.4 children. Practicing Protestant women have generally lower fertility levels compared with their Catholics counterparts in Britain, although their fertility rates remain rather stable at around replacement level. In contrast to that, nominal Protestant women experienced a decline from 2.3 children to 1.8 children, converging with the fertility level of the non-affiliated women. Thus, fertility differences among religious groups in Britain appear to have narrowed, although Catholic women, whether practicing or nominal, still show distinctly higher fertility compared to other women.

In France, on the other hand, there is a clearer pattern of fertility divergence between practicing Catholic women and non-practicing ones, whether they are religiously affiliated or not. Following an initial decline from an average of 2.7 children, fertility of younger cohorts of practicing Catholic women rose back again to a level of 2.6, while fertility of nominal Catholic and non-affiliated women continued to decline to a level of 1.9 and 1.7, respectively (Fig. 3b). A similar decline (from an average of 2.9 children) followed by a rise in fertility has also occurred among practicing Catholic women in the Netherlands (Fig. 3c), although fertility has not returned to its initial level and the average family size for practicing Catholics in the youngest cohort is 2.4. By contrast, fertility among nominally Catholic women declined from 2.6 to 1.8 children on average.

Interestingly, practicing Dutch Reformed women experienced a substantial increase in fertility from 2.4 to three children on average, while fertility of nominal Dutch Reformed, which was initially at a similar level, declined to 2.2 children. A divergence has also occurred among practicing and nominal conservative Protestants, as the former maintained relatively high fertility levels (although with a decline from 3.5 to 2.6), while the latter experienced a decline from three to less than two children on average.

Although each country shows somewhat different trends, a general pattern emerges where practicing religious women either maintain high fertility levels or even experience an increase in family size, while non-practicing and non-affiliated women experienced a decline in fertility from one cohort to another (with the exception of nominal Catholic women in Britain). Moreover, women who state having no religion consistently show the lowest fertility compared with all other groups. These findings give some support for the research hypotheses on religious differences in fertility and the increasing fertility gap by religious practice.

The following section describes the results of a multivariate regression model for completed fertility, controlling for religious group (combined affiliation and practice), birth cohort, level of education and nativity.

### Multivariate Regression Analysis

Table 1 presents the results of an OLS multivariate regression model for the number of children ever born to women aged 40 and above. The first model in each country is the basic model for the relationship between religious group and fertility, including other covariates, while the second model introduces interaction terms between religious group and birth cohort.^7^ The predicted means for the average number of children by religious group and cohort are shown in “Appendix,” Table 6a–c.

**Table 1:** OLS regression results: children ever born for women aged 40–79 in Britain, France and the Netherlands

	Britain		France		The Netherlands	
	Model 1	Model 2	Model 1	Model 2	Model 1	Model 2
Religious group^a^						
No religion	Ref	Ref	−0.159	−0.078	Ref	Ref
Nominal Catholic	0.320***	0.683**	Ref	Ref	0.174***	0.294
Practicing Catholic	0.660***	1.565***	0.227***	0.127	0.517***	0.681***
Nominal Protestant	0.134**	0.402***			0.411***	0.788*
Practicing Protestant	0.181**	0.208			1.427***	1.311***
Nominal Dutch R′					0.358***	0.056
Practicing Dutch R′					0.999***	0.166
Birth cohort						
1930–1939	Ref	Ref	Ref	Ref	Ref	Ref
1940–1949	−0.040	0.238	−0.419***	−0.402***	−0.312***	−0.384***
1950–1959	−0.111	0.148	−0.426***	−0.484***	−0.265***	−0.411***
1960–1965	−0.090	0.103	−0.413***	−0.448***	−0.356***	−0.420***
Education						
Lower secondary	0.443***	0.419***	0.332***	0.336***	0.131**	0.112*
Upper secondary	Ref	Ref	Ref	Ref	Ref	Ref
Tertiary	−0.042	−0.050	−0.028	−0.037	−0.298***	−0.302***
Foreign born	−0.131	−0.132	0.219**	0.204**	0.090	0.103
No religion × cohort						
1940–1949				−0.115		
1950–1959				0.056		
1960–1965				−0.247		
Nominal Cath’ × cohort						
1940–1949		−0.460				−0.275
1950–1959		−0.692**				−0.019
1960–1965		0.005				−0.160
Practicing Cath’ × cohort						
1940–1949		−1.140***		−0.124		−0.378
1950–1959		−1.371***		0.337*		−0.361
1960–1965		−0.893**		0.420*		−0.012
Nominal Prot’ × cohort^b^						
1940–1949		−0.318*				−0.328
1950–1959		−0.330*				−0.391
1960–1965		−0.348*				−0.607
Practicing Prot’ × cohort						
1940–1949		−0.177				0.076
1950–1959		0.268				0.584**
1960–1965		0.107				−0.349
Nominal Dutch R × cohort						
1940–1949						0.342
1950–1959						0.342
1960–1965						0.473
Practicing Dutch R × cohort						
1940–1949						1.536***
1950–1959						0.947***
1960–1965						1.087***
Constant	1.827***	1.625***	2.289***	2.314***	2.080***	2.177***
*N*	2760	2760	2837	2837	2431	2431
Adjusted *R* ^2^	0.04	0.05	0.05	0.05	0.14	0.15

* *p* < 0.1; ** *p* < 0.05; *** *p* < 0.01

^a^ *Nominal* attending religious services less than once a month. *Practicing* attending religious services once a month or more

^b^In the Netherlands, “Protestant” refers to Calvinist and Evangelical denominations

The results of the first model show that women who are either practicing or nominally affiliated with a particular religion have significantly higher fertility compared with women who profess to no religion. The exception to that is in France, where no significant difference was found between non-affiliated and nominally Catholic women. This may be due to the particularly large proportion of the latter in France which may be highly heterogeneous in terms of loyalty to Catholic norms about reproduction.

The findings from the second model of the regression, which includes the interaction term between religious group and birth cohort, show some contrasting trends in each country. In Britain, although the main effect for practicing Catholic women remains strongly positive, the interaction between practicing Catholics and birth cohort is significantly negative in reference to the oldest cohort group, reflecting the sharp decline in fertility to levels that are closer to the non-affiliated. Significant negative interaction is also found for nominal Catholics from the 1950–1959 cohort and for nominal Protestants. Thus, contrary to the third hypothesis about increasing fertility differences by religiosity, the results from Britain point to a convergence of family size between nominally and practicing religious women and their non-affiliated counterparts. Nevertheless, as can be seen by the predicted means of children ever born (Table 6a–c), the gap between non-affiliated and Catholic women (practicing or nominal) increases again in the most recent cohort (1960–1965). Therefore, the apparent fertility decline among Catholic women may be temporary. On the other hand, in France and the Netherlands there is evidence of increasing divergence between non-affiliated and practicing religious women: In France, this is shown by the positive interaction effect among Practicing Catholics in the two recent cohorts. In the Netherlands, a positive interaction term is found for practicing Dutch Reformed in all cohorts compared to the earliest one. Among practicing conservative Protestants, the interaction is only significant for the 1950–1959 cohort, while no interaction effect is found for practicing Catholics.

The increasing fertility among practicing Dutch Reformed in relation to their non-affiliated counterparts could be attributed to the growing proportion of the fundamental wing in this group, as a result of their stronger attachment to the church compared with liberal members who are more likely to reduce their attendance or leave their faith altogether (Knippenberg 1998, 2005).

The results of the OLS regression and the predicted means for children ever born give further support to the first two hypotheses: across cohorts, practicing religious women have the highest fertility levels compared with other groups, while non-affiliated women are consistently at the lower end of the scale. Fertility levels of nominally religious women are generally lower than that of practicing ones, although in most cases they have significantly higher fertility than non-affiliated women. The third hypothesis, which implies an increasing fertility gap between practicing and non-practicing women among the younger cohorts, is only partially supported by the results. However, these findings are limited to women who have already completed their births. The following analysis follows religious differences in the transition to first birth by reconstructing women’s birth histories from the age of fifteen.

### Event History Analysis of the Transition to First Birth

Table 2a–c summarizes the main findings from the reconstructed birth histories of women born before and after 1960 by religious group. It is shown that while the median age at first birth has increased for all women who were born after 1960, the proportion of childless women among the actively religious has decreased in all three countries. By contrast, non-affiliated women within the post-1960 cohorts have the highest proportion of childlessness and nominal religious women are usually found in between. In addition, the proportion of women who have had their first birth outside marriage has increased substantially for all women, although the largest increase was among the non-affiliated. In both cohort groups, non-affiliated women have the highest prevalence of births outside marriage, except among the pre-1960 cohorts in Britain, where nominal Catholic women show a higher percentage of first births out of wedlock (17 % compared to 11 % among the non-affiliated). This may be the consequence of socioeconomic differences between Catholics and other population groups in Britain (O’Grada and Walsh 1995).

**Table 2:** Median age at first birth, proportion childless and first births outside marriage by religious group for women aged 15–45

Religious Group^a^	Pre-1960 cohorts				Post-1960 cohorts			
	Median age at first birth	% Childless at age 45	% births outside marriage	*N* women	Median age at first birth	% Childless at age 45	% births outside marriage	*N* women
a. Britain								
No religion	25	13	11	562	27	15	52	1246
Nominal Catholic	25	10	17	116	27	11	47	131
Practicing Catholic	26	14	1	93	29	3	37	66
Nominal Protestant	25	12	8	898	28	12	33	517
Practicing Protestant	27	13	5	301	29	6	21	101
Total	25	12	8	1970	27	13	43	2061
b. France								
No religion	24	15	26	137	27	20	63	387
Nominal Catholic	25	13	16	1934	28	14	43	1826
Practicing Catholic	26	17	13	449	27	3	33	161
Total	25	14	16	2520	28	14	45	2374
c. Netherlands								
No religion	28	19	6	411	31	23	22	662
Nominal Catholic	27	14	2	252	30	14	13	364
Practicing Catholic	28	22	1	141	29	13	9	45
Nominal Protestant	27	17	2	35	30	18	12	33
Practicing Protestant	25	10	4	116	27	12	2	112
Nominal Dutch R′	27	18	1	123	29	7	9	84
Practicing Dutch R′	25	20	0	86	29	9	4	53
Total	27	17	3	1164	30	18	15	1353

^a^See comment “a” in Table 1

As expected, these findings point to more traditional family behaviors among women with stronger attachment to religion. In addition, religious differences in childlessness and births out of wedlock have become more marked for the post-1960 cohorts. These findings therefore support the fourth hypothesis about the higher propensity of the more religious women to enter motherhood and the increasing gap in this propensity among religious groups from recent cohorts. The significance of these differences, controlling for other socio-demographic factors, is further explored in the logistic regression analysis, which is presented in Table 3.

**Table 3:** Discrete-time event history analysis for first birth among women born prior and after 1960 in Britain, France and the Netherlands

	Britain				France				Netherlands			
	<1960		≥1960		<1960		≥1960		<1960		≥1960	
	Model 1	Model 2	Model 1	Model 2	Model 1	Model 2	Model 1	Model 2	Model 1	Model 2	Model 1	Model 2
Age	2.942***	1.419***	1.786***	1.237***	3.475***	2.002***	3.292***	1.921***	4.311***	1.891***	4.076***	2.088***
Age squared	0.981***	0.992***	0.990***	0.995***	0.977***	0.986***	0.980***	0.989***	0.975***	0.988***	0.978***	0.988***
Married		1.000		1.000		1.000		1.000		1.000		1.000
Single		0.028***		0.080***		0.054***		0.033***		0.013***		0.019***
In cohabitation		0.281***		0.352***		0.326***		0.338***		0.178***		0.173***
Divorced/Separated/Widowed		0.105***		0.164***		0.303***		0.127***		0.148***		0.070***
No religion	1.000	1.000	1.000	1.000	1.000	1.000	1.000	1.000	1.000	1.000	1.000	1.000
Nominal^a^ Catholic	1.175	1.423***	1.119	1.120	1.039	1.146	0.911	0.819**	1.122	1.142*	1.149*	0.959
Practicing Catholic	0.914	1.214	1.182	1.098	0.834	1.075	1.303**	1.026	0.949	1.254**	1.689***	1.223
Nominal Protestant	0.954	0.996	1.049	0.918					1.334*	1.441**	1.010	0.940
Practicing Protestant	0.874	0.966	1.116	0.980					1.625***	1.938***	2.537***	1.532***
Nominal Dutch R′									1.090	1.026	1.416**	1.157
Practicing Dutch R′									1.076	1.149	1.742***	0.990
Enrolled in the education system^b^	0.601***	0.793**	0.614***	0.754***	0.434***	0.627***	0.319***	0.489***				
Lower secondary	1.351***	1.289***	2.523***	2.512***	1.223***	1.070	1.342***	1.394***	1.423***	1.082	1.729***	1.533***
Upper secondary	1.000	1.000	1.000	1.000	1.000	1.000	1.000	1.000	1.000	1.000	1.000	1.000
Tertiary education	0.864**	0.950	0.680***	0.712***	0.870*	1.004	0.646***	0.682***	0.588***	0.803***	0.604***	0.745***
Foreign born	0.797	1.068	0.670**	0.657**	1.110	1.042	1.062	1.475***	1.176	1.439**	1.228	1.759***
Pseudo *R* ^2^	0.10	0.26	0.08	0.16	0.12	0.25	0.16	0.28	0.14	0.32	0.17	0.31
*N* (women years)	24,603		26,413		34,280		29,721		31,317		27,244	

The exposure period for the transition to first birth includes women aged 15–45. The model also controls for time period in 5 years intervals (not shown here)

^a^See comment “a” in Table 1

^b^Timing of enrollment is not available for the Netherlands

The results of the logistic regression model appear to vary from one country to another. In Britain for example, no significant difference is found in the likelihood of first birth for practicing Catholic or Protestant women in comparison with non-affiliated women. However, when controlling for marital status, nominal Catholic women in the pre-1960 cohorts show significantly higher likelihood of entering motherhood compared with the reference group (odds ratio of 1.423, *p* < 0.01). A similar trend is found in the Netherlands, where nominal and practicing Catholic women born before 1960 show significantly higher likelihood of experiencing the transition to first birth when marital status is controlled. This could be attributed to the tendency of late entry to marriage (as well as later transition to first birth) among Catholics (Lehrer 2000, 2004b). According to Lehrer, the cost of marital dissolution for Catholics is especially high, due to the anti-divorce doctrine of the Catholic Church. Therefore, the search for a spouse will be longer for Catholics than for other religious groups (ibid). This pattern, however, is only evident among earlier cohorts. For Catholic women born after 1960 in the Netherlands, as well as among Dutch Reformed, the higher transition rates to first birth are insignificant once marital status is included in the model. Similarly, among women born after 1960 in France, the coefficient for practicing Catholic changes from significantly positive to insignificant when controlling for marital status and a negative relationship emerges for nominal Catholic women. This may be the result of differential marriage trends among religious groups in these countries; while the proportion of ever married people in Europe has declined over time (Perelli-Harris et al. 2009), the more religious segments of society are more likely to maintain high marriage rates (Régnier-Loilier and Prioux 2008). Lehrer (2004b) has argued that the stronger marriage preferences among religious women are closely linked to fertility aspirations. Thus, women who expect to have a large family would be more inclined to marry than to live in non-marital cohabitation, which is considered to be a less stable union. Therefore, the higher transition rates to first birth among more religious women in recent cohorts can in some cases be attributed to differences in marriage patterns.

The group that shows consistently higher likelihood of experiencing first birth compared with non-affiliated women among both earlier and recent cohorts is practicing conservative Protestant women in the Netherlands. On the other hand, for nominal women from this group, the higher likelihood of first birth compared with women with no religion is only evident among earlier cohorts.

Thus, while the findings from Table 2a–c support the fourth hypothesis on the increasing gap in the transition to first birth by level of religiosity, the results of the multivariate logistic regression are not consistent across countries. This is partly due to differences in the timing of first birth, as in some religious denominations women tend to start childbearing at a later age and are more likely to defer the first birth until after they are formally married. In addition, as past studies show, more religious women have higher marriage rates compared with others, which are also related to higher likelihood of entering motherhood. Indeed, the results of the logistic regression show that in all three countries, current marital status is strongly linked to the transition to first birth, as the odds for entering motherhood are considerably lower for single women and those in non-marital cohabitation compared with that of married women.

Another noteworthy finding is that in all countries, the highest level of education achieved as well as the time-varying covariate of enrollment in education is associated with lower propensity to experience the transition to first birth. In addition, a significant negative effect on first birth is found in Britain for those who were born abroad, while in France and in the Netherlands the effect is positive. This is likely to be the result of differences in the sending countries from which people immigrate to each of these destinations. For example, in recent decades a large proportion of immigrants to Britain have come from Eastern Europe, where fertility levels are often lower in relation to Britain (Coleman and Dubuc 2010). On the other hand, the majority of immigrants in France and the Netherlands arrived from countries that are characterized with relatively high fertility.^8^ It should be noted, though, that if the analysis included women from non-Christian religions, the results for the effect of nativity might have been more strongly positive, since immigrants to Europe from Muslim countries tend to have higher fertility compared with the native population (Kaufmann et al. 2012).

Finally, a robustness check was made using different cutoff points (1955 and 1965) between the cohorts. In general, the change in the cutoff point did not yield very different results, except in the case of Britain, where the odds for the transition to first birth among nominal and practicing Catholic women who were born after 1965 were significantly higher compared to non-affiliated women, ceteris paribus (not shown). This trend is similar to the findings on completed family size (see Fig. 3a), where the fall in Catholic fertility is followed by a rise among the 1960–1965 cohort. Therefore, this may represent a trend of resurgence to higher fertility levels within that group.

### Religiosity as a Determinant of Progression to Higher Parity

So far, the presented findings were based on retrospective data analysis, in which religiosity is measured after the time of childbearing. This section includes findings from a longitudinal analysis of the relationship between religiosity and subsequent childbearing. Table 4 presents the results of the logistic regression model for the likelihood of experiencing an additional childbirth within 3 years from the first observation. The findings from Britain show no relationship between religious indicators and the likelihood of having an additional birth within the observed time period, which is in accordance with earlier findings on fertility convergence between religious groups there. By contrast, practicing Catholic women in France show higher likelihood of progressing to higher parity compared with non-affiliated women (odds ratio of 2.421, *p* < 0.1).

**Table 4:** Logistic regression estimates for additional childbirth within 3 years for women aged 20–42 in Britain, France and the Netherlands. The models for France and the Netherlands include a propensity score for attrition (see "Appendix")

	Britain	France	The Netherlands
Religious group^a^			
No religion	1.000	1.000	1.000
Nominal Catholic	0.680	1.084	1.794**
Practicing Catholic	0.978	2.421*	0.903
Nominal Protestant	1.070		2.404
Practicing Protestant	1.047		3.017**
Nominal Dutch R′			1.959
Practicing Dutch R′			0.953
Age			
Age	1.764***	1.183	1.731
Age squared	0.987***	0.995	0.987**
Marital status			
Married	1.000	1.000	1.000
Cohabiting	0.866	0.552**	0.864
Single	0.749	0.145***	0.253**
Education			
Lower secondary	1.705**	1.426	0.982
Upper secondary	1.000	1.000	1.000
Tertiary	1.513**	1.478	2.691**
Employment status			
In paid employment	0.922	0.573*	0.975
Nativity			
Foreign born	1.364	1.269	2.594
Age at first birth	1.109**	0.974	1.153**
Birth interval			
3–5 years	1.000	1.000	1.000
5–7 years	1.086	1.682*	1.232
7–10 years	0.824	0.573	0.730
10+ years	0.736	0.293*	0.473
Parity			
1 child	1.000	1.000	1.000
2 children	0.359***	0.168***	0.332***
3+ children	0.462**	0.088***	0.298***
*N*	1299	817	822
Pseudo-*R* ^2^	0.25	0.33	0.35

* *p* < 0.1; ** *p* < 0.05; *** *p* < 0.01

^a^See comment “a” in Table 1

In the Netherlands, a positive effect on parity progression was found among nominal Catholics (odds ratio of 1.794, *p* < 0.05) and conservative Protestant women who attend services regularly (odds ratio of 3.017, *p* < 0.05) compared with their non-affiliated peers. No significant effect was found among the other religious groups, although this may be due to small sample sizes; when Dutch Reformed are grouped together with conservative Protestant women, both nominal and practicing women have a significantly higher likelihood of progressing to higher parity compared with non-affiliated women (not shown). Therefore, in the Netherlands fertility patterns not only differ by religious practice, but also nominally religious women are more likely to progress to higher parity compared with non-affiliated ones. These patterns may be related to the pronounced religious diversity in the Netherlands and the fact that religion was considered an important aspect of social identity, at least during the pillarization period (Knippenberg 1998; van Poppel 1985; van Rooden 2003).

## Summary and Discussion

This study examined the changing relationship between religion and fertility in three countries that are considered to be highly secularized. While institutionalized forms of religion have been declining in Western Europe from one generation to the next, religion continues to be an important determinant of fertility and family formation patterns in this region. By reconstructing women’s births histories, it was possible to detect differences in fertility trends by religious affiliation and practice between countries and across cohorts. Moreover, these differences are confirmed in a longitudinal analysis of the role of religion in determining future childbearing.

In accordance with the study’s first two hypotheses, both active and nominal religious adherence is linked with higher fertility levels, where the highest levels are among practicing religious women. The third hypothesis, which implies a growing fertility divergence by religiosity among younger cohorts, is only partially supported by the results; an increasing fertility gap is found between practicing Catholics and non-practicing women in France as well as between practicing Protestants (Dutch Reformed and others) and non-practicing women in the Netherlands. On the other hand, fertility differences between practicing Catholic and other women in Britain have narrowed, although an increasing divergence is evident for the most recent cohort. The fourth hypothesis about the higher propensity of more religious women to enter motherhood and the increase in that gap among recent cohorts received some support in this study; the proportion of childless women has increased the most among non-affiliated women, while it has generally declined for the practicing religious ones. However, these differences are at least partly attributed to differential marriage patterns among religious groups.

Since the relationship between religious indicators and fertility behavior varies across countries, the argument of increasing selectivity among religiously observant women is not sufficient to understand these trends. Rather, additional country-specific circumstances should be taken into account. The persistence of fertility differences by religion in France and the Netherlands may be related to social conflicts on the basis of religious identity in these countries. In the case of France, the ongoing debate about its character as a secular country and anti-Church sentiments may account for the stronger divergence between actively religious and non-practicing individuals. In the Netherlands, as a result of the historical pillarization system, religious affiliation may still constitute an important part of social identity, which could account for the differential fertility patterns by religiosity. In Britain, on the other hand, religion appears to play a less significant role in explaining fertility differences in recent cohorts. It should be noted, though, that the apparent convergence in fertility patterns between Catholic women (practicing or nominal) and non-affiliated women in Britain may be temporary, as there is evidence for an upward trend in completed fertility among Catholic women who have just recently completed their childbearing years.

Overall, it appears that, as noted by Greeley (2003), there is no one single process of secularization in Europe. Although the general direction appears to be one of decline in indicators of religiosity, each society has its own variation of this process and shows different patterns of continuity and change in the extent to which religion influences individual behavior. Future research of the relationships between religion and fertility should pay attention to the variety of secularization processes in Europe and the way these influence the changing role of religion in shaping fertility patterns.

## Appendix: Correcting for Attrition in the GGP Data from France and the Netherlands

Out of the 10,079 respondents who took part in the French GGP survey in 2005, only 6534 people also participated in the second wave in 2008. The main reasons for the attrition include contact loss, refusal and in some cases death. Since the attrition was not due to random causes, the sample interviewed in 2008 is not representative of the French population in that year. However, the data can be used in a longitudinal approach, provided that appropriate weighting is applied to correct for attrition (see: Régnier-Loilier et al. 2011). Based on a method proposed by Kristman et al. (2005), a propensity score was calculated by modeling the probability of attrition (yes or no) as a function of the relevant predictor variables that were used in the longitudinal analysis (age, education, religious affiliation and practice, marital status, number of children, country of birth and employment status). After running a logistic regression model that included all these variables, the predicted probability of attrition for each respondent was then included as a controlling variable in the logistic regression model for parity progression (Table 4).

The attrition rate in the GGP survey from the Netherlands was somewhat lower; out of 8161 respondents who were interviewed in the first wave in 2002–2004, 6091 people were re-interviewed in the second wave in 2006–2007. While the attrition rate is not as high as in France, it is still substantial (about 25 %) and may cause distortion of the results. Therefore, the same correction method was applied in the longitudinal analysis from the Netherlands (Table 4).

**Table 5:** Sample sizes of religious groups by sex and birth cohort

	Birth cohort					
	1930–1939	1940–1949	1950–1959	1960–1969	1970–1979	1930–1979
a. Britain, women						
No religion	126 (20 %)	214 (24 %)	387 (39 %)	574 (47 %)	577 (55 %)	1878 (39 %)
Nominal Catholic	23 (4 %)	45 (5 %)	57 (6 %)	80 (6 %)	50 (5 %)	260 (5 %)
Practicing Catholic	35 (5 %)	36 (4 %)	34 (4 %)	47 (4 %)	34 (3 %)	191 (4 %)
Nominal Protestant	247 (40 %)	387 (43 %)	336 (33 %)	338 (27 %)	228 (22 %)	1536 (32 %)
Practicing Protestant	130 (21 %)	118 (13 %)	86 (9 %)	68 (6 %)	52 (5 %)	454 (10 %)
Other	61 (10 %)	101 (11 %)	93 (9 %)	126 (10 %)	108 (10 %)	487 (10 %)
Total	622 (100 %)	901 (100 %)	1003 (100 %)	1233 (100 %)	1047 (100 %)	4806 (100 %)
b. Britain, men						
No religion	174 (35 %)	309 (41 %)	434 (52 %)	578 (57 %)	621 (69 %)	2116 (53 %)
Nominal Catholic	14 (3 %)	34 (5 %)	49 (6 %)	61 (6 %)	41 (4 %)	199 (5 %)
Practicing Catholic	20 (4 %)	21 (3 %)	14 (2 %)	25 (3 %)	14 (2 %)	94 (2 %)
Nominal Protestant	189 (38 %)	278 (37 %)	232 (28 %)	225 (22 %)	122 (13 %)	1046 (26 %)
Practicing Protestant	54 (11 %)	55 (7 %)	44 (5 %)	40 (4 %)	17 (2 %)	210 (5 %)
Other	44 (9 %)	55 (7 %)	61 (7 %)	83 (8 %)	92 (10 %)	336 (9 %)
Total	495 (100 %)	752 (100 %)	835 (100 %)	1012 (100 %)	907 (100 %)	4001 (100 %)
c. France, women						
No religion	24 (4 %)	33 (4 %)	76 (8 %)	101 (10 %)	144 (16 %)	378 (9 %)
Nominal Catholic	401 (65 %)	607 (75 %)	789 (78 %)	744 (73 %)	631 (70 %)	3172 (72 %)
Practicing Catholic	172 (28 %)	128 (16 %)	82 (8 %)	89 (9 %)	49 (5 %)	520 (12 %)
Other	22 (3 %)	41 (5 %)	65 (6 %)	86 (8 %)	84 (9 %)	298 (7 %)
Total	619 (100 %)	809 (100 %)	1012 (100 %)	1020 (100 %)	908 (100 %)	4368 (100 %)
d. France, men						
No religion	27 (5 %)	42 (6 %)	67 (9 %)	108 (14 %)	124 (18 %)	368 (11 %)
Nominal Catholic	390 (77 %)	553 (80 %)	577 (77 %)	597 (74 %)	466 (66 %)	2583 (75 %)
Practicing Catholic	69 (14 %)	58 (8 %)	28 (4 %)	38 (5 %)	18 (3 %)	211 (6 %)
Other	22 (4 %)	44 (6 %)	72 (10 %)	59 (7 %)	93 (13 %)	290 (8 %)
Total	508 (100 %)	697 (100 %)	744 (100 %)	802 (100 %)	701 (100 %)	3452 (100 %)
e. Netherlands, women						
No religion	124 (27 %)	243 (36 %)	382 (47 %)	503 (47 %)	374 (50 %)	1626 (43 %)
Nominal Catholic	76 (16 %)	155 (23 %)	190 (23 %)	289 (27 %)	173 (23 %)	883 (23 %)
Practicing Catholic	82 (18 %)	68 (10 %)	43 (5 %)	38 (4 %)	10 (1 %)	241 (6 %)
Nominal Protestant	10 (2 %)	24 (4 %)	22 (3 %)	28 (3 %)	20 (3 %)	104 (3 %)
Practicing Protestant	51 (11 %)	51 (8 %)	50 (6 %)	66 (6 %)	52 (7 %)	270 (7 %)
Nominal Dutch R′	57 (12 %)	69 (10 %)	60 (7 %)	55 (5 %)	46 (6 %)	287 (8 %)
Practicing Dutch R′	45 (10 %)	30 (4 %)	28 (3 %)	30 (3 %)	16 (2 %)	149 (4 %)
Other	18 (4 %)	39 (6 %)	43 (5 %)	66 (6 %)	51 (7 %)	217 (6 %)
Total	463 (100 %)	679 (100 %)	818 (100 %)	1075 (100 %)	742 (100 %)	3777 (100 %)
f. Netherlands, men						
No religion	113 (30 %)	203 (36 %)	329 (49 %)	351 (54 %)	275 (59 %)	1271 (47 %)
Nominal Catholic	90 (24 %)	154 (27 %)	158 (23 %)	141 (22 %)	78 (17 %)	621 (23 %)
Practicing Catholic	64 (17 %)	59 (10 %)	29 (4 %)	20 (3 %)	6 (1 %)	178 (7 %)
Nominal Protestant	8 (2 %)	17 (3 %)	18 (3 %)	12 (2 %)	11 (2 %)	66 (2 %)
Practicing Protestant	22 (5 %)	37 (7 %)	33 (5 %)	40 (6 %)	29 (6 %)	161 (6 %)
Nominal Dutch R′	35 (9 %)	51 (9 %)	59 (9 %)	30 (5 %)	16 (3 %)	191 (7 %)
Practicing Dutch R′	23 (6 %)	21 (4 %)	20 (3 %)	18 (3 %)	10 (2 %)	92 (3 %)
Other	18 (5 %)	25 (4 %)	32 (5 %)	37 (6 %)	40 (9 %)	152 (6 %)
Total	373 (100 %)	567 (100 %)	678 (100 %)	649 (100 %)	465 (100 %)	2732 (100 %)

**Table 6:** Predicted means for the number of children ever born (based on the OLS regression presented in Table 1)

Cohort	No religion	Nominal Catholic	Practicing Catholic	Nominal Protestant	Practicing Protestant
a. Britain					
1930–1939	1.7	2.4	3.3	2.1	2.0
1940–1949	2.0	2.2	2.4	2.1	2.0
1950–1959	1.9	1.9	2.1	2.0	2.4
1960–1965	1.8	2.5	2.5	1.9	2.2

Cohort	No religion	Nominal Catholic	Practicing Catholic
b. France			
1930–1939	2.4	2.5	2.6
1940–1949	1.9	2.1	2.1
1950–1959	2.0	2.0	2.5
1960–1965	1.7	2.0	2.6

Cohort	No religion	Nominal Catholic	Practicing Catholic	Nominal Protestant	Practicing Protestant	Nominal Dutch R′	Practicing Dutch R′
c. Netherlands							
1930–1939	2.1	2.4	2.8	2.9	3.5	2.2	2.3
1940–1949	1.8	1.8	2.1	2.2	3.1	2.2	3.5
1950–1959	1.7	2.0	2.1	2.1	3.6	2.1	2.8
1960–1965	1.7	1.9	2.4	1.9	2.7	2.3	3.0

Group-specific means are used for education

## Footnotes

1 The proportion of people attending religious services on a weekly basis is slightly lower and is estimated at 9–10 % in all three countries for the years 2008–2009 (Pew Research Center 2013; Statistics Netherlands 2012; Voas and Ling 2010). However, the decline in religious participation over the past decades has been particularly steep in the Netherlands: during the 1970s more than 40 % of the Dutch population attended services at least once a week compared with around 20 % of the population in Britain and France (Norris and Inglehart 2004).

2 Regular church attendance in Knippenberg (2005) refers to once every two weeks or more.

3 For a more detailed discussion on the religion question in France, see Régnier-Loilier and Prioux (2008).

4 Only biological children are included.

5 Additional robustness checks were conducted using 1955 and 1965 as the threshold years for earlier and recent cohorts.

6 Before 2008, the last time the BHPS included a question on religious affiliation is in 2004.

7 Due to small sample size of the no religion group in France, nominal Catholic is used as the reference category. However, results are qualitatively the same when running the model with no religion as the reference category.

8 The main source of immigration to France is from African countries and mainly from Northern Africa (Davie 2007). In the Netherlands, a large proportion of immigrants have arrived from the former Dutch colony of Surinam (Knippenberg 2005).
